# Comparison of Intraoperative and Postoperative Outcomes of Laparoscopic and Open Myomectomy in Women With Uterine Fibroids: A Retrospective Study at a Tertiary Care Hospital in Central India

**DOI:** 10.7759/cureus.91211

**Published:** 2025-08-28

**Authors:** Nidhi Agarwal, Anshika Agarwal, Rubina Dohare

**Affiliations:** 1 Obstetrics and Gynaecology, Government Medical College, Datia, Datia, IND

**Keywords:** minimally invasive surgery, myomectomy, retrospective study, surgical outcome, uterine fibroids

## Abstract

Background

Myomectomy is a uterus-preserving surgery for women with symptomatic uterine fibroids desiring fertility. Comparing laparoscopic myomectomy (LM) and open myomectomy (OM) can help guide optimal surgical decisions. This study aimed (1) to compare intraoperative and postoperative outcomes between LM and OM, and (2) to identify clinical predictors influencing the choice of surgical approach in women with symptomatic uterine fibroids.

Methodology

This prospective, comparative, non-randomized study included 80 women with symptomatic fibroids, consecutively sampled and allocated into LM (n = 40) and OM (n = 40) groups based on fibroid size, number, and surgeon decision. Outcomes included intraoperative blood loss, operative time, pain scores (Numeric Rating Scale (NRS)), hospital stay, and time to resume normal activities. Data were analyzed using t-tests, linear and logistic regression, and repeated measures analysis of variance. Surgeons performing LM and OM had comparable experience, and outcome assessors were blinded to surgical groups.

Results

Mean fibroid size was significantly larger in the OM group (7.61 ± 1.32 cm) than in the LM group (4.81 ± 1.10 cm; p < 0.001). OM had greater intraoperative blood loss (278.25 ± 57.87 mL vs. 112.38 ± 33.29 mL; p < 0.001) and higher postoperative pain scores (6.70 ± 0.82 vs. 2.42 ± 0.93; p < 0.001), but shorter operative time (98.03 ± 15.67 vs. 133.47 ± 15.96 minutes; p < 0.001). LM patients had shorter hospital stays (2.08 ± 0.47 vs. 5.30 ± 1.27 days; p < 0.001) and resumed normal activities earlier (8.38 ± 2.26 vs. 16.10 ± 4.02 days; p < 0.001). Missing data were minimal (<2%) and handled using listwise deletion. All measurement instruments (e.g., NRS) were validated tools with cited references.

Conclusions

LM provides superior short-term outcomes, namely, less pain, lower blood loss, and shorter recovery, despite longer operative time. Surgical planning should consider fibroid size, surgeon expertise, and patient preference. Further prospective studies are needed for long-term outcome assessment.

## Introduction

Uterine fibroids, or leiomyomas, are the most common benign tumors in women of reproductive age, affecting up to 70% of women by age 50 [1]. These smooth muscle growths can cause heavy menstrual bleeding, pelvic pain, and infertility, significantly impairing quality of life [2]. Myomectomy is the preferred treatment for symptom relief in women seeking uterine preservation and future fertility [3]. Open myomectomy (OM) involves a large abdominal incision, while laparoscopic myomectomy (LM) uses smaller incisions and specialized tools, reducing tissue trauma [4].

Evidence suggests LM offers several advantages over OM. Studies have shown that LM is associated with reduced intraoperative blood loss, shorter hospital stays, and quicker recovery [4]. Andrews et al. reported lower transfusion needs and complication rates with LM, particularly in women with multiple fibroids [5]. Factors such as fibroid size, location, and operative time increase postoperative risk, with OM often being an independent risk factor [6]. LM also reduces fibroid recurrence (10.2% vs. 23.8%) and cesarean delivery rates, while maintaining reproductive outcomes comparable to OM [7]. Single-port LM studies further support its safety and favorable outcomes [8].

However, Wise et al. found no significant difference in pregnancy or live-birth rates between OM, LM, and hysteroscopic myomectomy, emphasizing the influence of individual patient and fibroid characteristics [9]. Access to LM is improving, as shown by Kim et al., who found equitable rates among racial groups after adjusting for fibroid burden [10]. Robotic-assisted LM has also shown potential to further reduce blood loss and conversion rates [11,12].

Given the high prevalence of uterine fibroids and the crucial role of myomectomy in their management, it is essential to refine surgical strategies. This study aimed to compare intraoperative and postoperative outcomes between LM and OM and to identify clinical predictors influencing the choice of surgical approach in women with symptomatic uterine fibroids.

## Materials and methods

Study design and setting

This prospective, comparative, non-randomized observational study was conducted in the Department of Obstetrics and Gynaecology, Government Medical College, Datia, India, between July 2023 and July 2024.

Study population and group allocation

A total of 80 women aged 18-45 years with symptomatic uterine fibroids were consecutively enrolled after meeting eligibility criteria. Participants were divided into the following two groups: laparoscopic myomectomy (LM, n = 40) and open myomectomy (OM, n = 40). Group allocation was non-randomized and based on fibroid characteristics such as size, number, and location, along with surgeon expertise and patient preference. The upper age limit of 45 years was chosen because myomectomy is primarily performed for fertility preservation; women above this age more commonly undergo hysterectomy or medical therapy rather than myomectomy, thus limiting generalizability in this subgroup.

Sample size justification

A minimum of 35 participants per group was required to achieve 80% power at an α of 0.05 to detect a medium effect size in the primary outcomes. To account for potential exclusions and ensure adequate power for repeated-measures analyses, 40 participants per group were included.

Inclusion and exclusion criteria

Women aged 18-45 years with symptomatic uterine fibroids confirmed by ultrasonography or MRI who provided written informed consent were included. Exclusion criteria were pregnancy, suspected or confirmed uterine malignancy or sarcoma, history of major pelvic surgery, contraindications to surgery or anesthesia such as coagulopathy, and unwillingness to participate.

Surgical procedures

All surgeries were performed by senior gynecological surgeons with more than 10 years of experience in both LM and OM to ensure standardized surgical techniques and minimize variability in surgical outcomes. The potential limitation regarding generalizability to less experienced surgeons is acknowledged in the Discussion section.

Data collection and outcome measures

Preoperative evaluation included history, clinical examination, laboratory investigations, and imaging. The primary outcomes were operative time in minutes measured from incision to skin closure, intraoperative blood loss in milliliters measured as suction volume minus irrigation plus gauze weight difference, postoperative pain assessed using the Numeric Rating Scale (NRS), hospital stay in days from surgery to discharge, time to resume normal activities in days based on patient self-report, and postoperative complications classified. The NRS is an 11-point scale ranging from 0, indicating no pain, to 10, indicating the worst imaginable pain, developed at McGill University, Montreal, Canada, and widely validated for postoperative pain assessment [13]. Pain scores were recorded at one, two, and four weeks postoperatively by trained nursing staff blinded to the surgical approach.

Ethical considerations

The study was approved by the Institutional Ethics Committee of Government Medical College, Datia (Approval No: 87/OBS/GMC/IECBMHR/2023). Written informed consent was obtained from all participants after explaining the study objectives, procedures, potential risks, and benefits. Confidentiality and anonymity of participant data were strictly maintained.

Statistical analysis

Data were analyzed using Jamovi version 2.3.28 (The Jamovi University, Sydney, Australia). Normality was assessed using the Shapiro-Wilk test. Parametric tests, including the independent t-test and repeated-measures analysis of variance, were applied to normally distributed data, while the Mann-Whitney U test and Friedman test were used for non-parametric data. Multivariate linear regression was used to identify predictors of postoperative pain, and binomial logistic regression was used to analyze factors influencing the choice of surgical approach. A p-value of less than 0.05 was considered statistically significant.

## Results

Patient demographics

The mean age of the patients undergoing OM was 34.06 ± 5.37 years, significantly higher than those undergoing LM, who had a mean age of 32.20 ± 4.38 years (p = 0.030). Patients in the OM group had significantly larger fibroids (7.61 ± 1.32 cm) compared to the LM group (4.81 ± 1.10 cm; p < 0.001) (Table 1).

**Table 1:** Comparison of demographic and intraoperative and postoperative parameters between the groups (N = 80). An independent t-test was used. Values are presented as mean ± standard deviation. *: p < 0.05; ***: p < 0.001

Variable	Open myomectomy (n = 40)	Laparoscopic myomectomy (n = 40)	t-statistic	P-value
Age (years)	34.06 ± 5.37	32.20 ± 4.38	-2.21	0.030*
Fibroid size (cm)	7.61 ± 1.32	4.81 ± 1.10	-10.3	<0.001***
Operative time (minutes)	98.03 ± 15.67	133.47 ± 15.96	10.02	<0.001***
Intraoperative blood loss (mL)	278.25 ± 57.87	112.38 ± 33.29	-15.71	<0.001***
Hospital stay (days)	5.30 ± 1.27	2.08 ± 0.47	-15.1	<0.001***
Postoperative pain score	6.70 ± 0.82	2.42 ± 0.93	-21.77	<0.001***
Return to normal activity (days)	16.10 ± 4.02	8.38 ± 2.26	-10.58	<0.001***

Intraoperative outcomes

The LM group had a significantly longer operative time (133.47 ± 15.96 minutes) compared to the OM group (98.03 ± 15.67 minutes; p < 0.001). However, intraoperative blood loss was significantly greater in the OM group, averaging 278.25 ± 57.87 mL versus 112.38 ± 33.29 mL in the LM group (p < 0.001) (Table 1).

Postoperative outcomes

Patients in the OM group had a significantly longer hospital stay (5.30 ± 1.27 days) than those in the LM group (2.08 ± 0.47 days; p < 0.001). Postoperative pain scores were markedly higher in the OM group (6.70 ± 0.82) compared to the LM group (2.42 ± 0.93; p < 0.001). Time to return to normal activity was also substantially delayed in OM patients (16.10 ± 4.02 days vs. 8.38 ± 2.26 days; p < 0.001) (Table 1).

Regression and predictive modeling

*Predictors of Postoperative Pain* 

OM independently predicted higher postoperative pain scores (β = 4.504; p < 0.001). Age showed a modest inverse association (β = −0.043; p = 0.041), indicating slightly lower pain levels in older patients. Other perioperative variables, including operative time, blood loss, and fibroid location, did not significantly predict pain (Table 2).

**Table 2:** Predictors of postoperative pain based on linear regression analysis. Model summary: R = 0.941; R² = 0.885; adjusted R² = 0.864. Outcome variable: immediate postoperative pain score (measured on the Numeric rating Scale). β estimates reflect the direction and magnitude of change in postoperative pain score per unit change in the predictor. ᵃ: Reference category used in dummy variable encoding. *: p < 0.05; ***: p < 0.001; NS: not significant (p > 0.05); OM: open myomectomy; LM: laparoscopic myomectomy

Predictor	Estimate (β)	Standard error (SE)	t-value	P-value
Interceptᵃ	3.517	1.346	2.613	0.011*
Postoperative complications				
Fever – none	0.403	0.31	1.298	0.199^NS^
Hematoma – none	0.194	0.483	0.402	0.689^NS^
Wound infection – none	-0.215	0.335	-0.641	0.523^NS^
Return to normal activity (days)	-0.029	0.032	-0.907	0.367^NS^
Hospital stay (days)	0.082	0.111	0.733	0.466^NS^
Operative time (minutes)	0.006	0.007	0.834	0.408^NS^
Intraoperative blood loss (mL)	-0.002	0.002	-0.874	0.385^NS^
Fibroid location				
Submucosal – intramural	0.52	0.27	1.925	0.058^NS^
Subserosal – intramural	-0.219	0.225	-0.973	0.334^NS^
Fibroid size (cm)	0.144	0.086	1.678	0.098^NS^
Group (OM vs. LM)	4.504	0.881	5.113	<0.001***
Age (years)	-0.043	0.021	-2.082	0.041*

Predictors of Surgical Approach

Fibroid size significantly predicted surgical method. Each 1 cm increase in diameter raised the odds of undergoing OM by over five times (odds ratio (OR) = 5.63; 95% confidence interval (CI) = 2.61-12.17; p < 0.001). Neither patient age nor fibroid location was a statistically significant predictor (Table 3).

**Table 3:** Predictors of odds of undergoing open myomectomy using a binomial logistic regression model. Model fit statistics: deviance = 47.5, AIC = 57.5, McFadden’s R² = 0.572. Binary outcome: groups = OM (open myomectomy) vs. LM (laparoscopic myomectomy). Estimates are log-odds; odds ratios >1 indicate an increased likelihood of OM. Reference category for fibroid location = intramural. Values are represented as estimate (β) ± SE **: p < 0.01; ***: p < 0.001; NS = not significant (p >0.05); SE: standard error; CI: confidence interval

Predictor	β ± SE	Z	OR	95% CI	P-value
Intercept	−14.703 ± 4.484	−3.279	4.12 × 10⁻⁷	6.28 × 10⁻¹¹–0.00270	0.001**
Age (years)	0.118 ± 0.0897	1.315	1.13	0.944–1.341	0.188^NS^
Submucosal vs. intramural	0.649 ± 1.0751	0.604	1.91	0.233–15.744	0.546^NS^
Subserosal vs. intramural	0.148 ± 0.8389	0.176	1.16	0.224–6.000	0.86^NS^
Fibroid size (cm)	1.729 ± 0.3932	4.397	5.63	2.607–12.172	<0.001***

Postoperative pain over time

Postoperative pain declined steadily (F = 18.044; p < 0.001), with a significantly faster reduction in the LM group (F = 7.888; p < 0.001). The type of surgery explained 75% of the variance in pain trajectory (F = 535.93; p < 0.001; η² = 0.747). Although minimal, age also had a statistically significant effect on pain trajectory (F = 4.14; p = 0.045) (Table 4).

**Table 4:** Improvement of postoperative pain score across time points. The repeated measures analysis of variance was used. RM factor: postoperative pain score (Numeric Rating Scale) measured at baseline, one, two, and four weeks. Effects of time, group, and their interaction on pain improvement were tested while controlling for age and other factors. Greenhouse-Geisser correction was applied if sphericity assumptions were violated. *: p < 0.05; ***: p < 0.001; NS: not significant (p > 0.05). F-values indicate the ratio of variance explained by each effect relative to error variance.

Effect	Sum of squares	Mean square	F	P-value
Within-subjects effects				
Improvement of postoperative pain over time	10.574	4.12	18.044	<0.001***
Time ✻ Group interaction	4.622	1.801	7.888	<0.001***
Time ✻ Age (years) interaction	0.404	0.157	0.689	0.538^NS^
Residual	45.121	0.228	—	—
Between-subjects effects				
Group	1,378.2	1,378.23	535.93	<0.001***
Age (years)	10.7	10.66	4.14	0.045*
Residual	198	2.57	—	—

## Discussion

Fibroid size emerged as a significant independent predictor for opting for OM, with an OR of 5.63 (p < 0.001). This observation is consistent with earlier studies demonstrating that larger fibroid volumes are associated with increased operative complexity, longer surgical durations, higher intraoperative blood loss, and prolonged hospital stays [14]. In our study, women in the OM group were slightly older and had markedly larger fibroids compared to those in the LM group (7.61 ± 1.32 cm vs. 4.81 ± 1.10 cm; p < 0.001). These findings reflect current surgical practice patterns in which open procedures are generally preferred for patients with larger or multiple fibroids to facilitate complete removal and minimize surgical complications.

Although fibroid location did not significantly influence surgical outcomes in our cohort, previous research has indicated that posteriorly located or deeply intramural fibroids can present technical challenges during laparoscopic procedures [12,15]. Consistent with existing literature, OM in our study was associated with significantly greater intraoperative blood loss compared to LM (278.25 ± 57.87 mL vs. 112.38 ± 33.29 mL; p < 0.001). Several studies have reported that adjunctive techniques such as temporary uterine artery clamping or preoperative vasopressin infiltration during LM can substantially reduce intraoperative blood loss without prolonging operative time [16]. Incorporating such methods into standard laparoscopic practice may further improve surgical outcomes, particularly in patients with moderate-sized fibroids.

Interestingly, despite the increased blood loss observed with OM, LM was associated with a significantly longer mean operative time (133.47 ± 15.96 minutes vs. 98.03 ± 15.67 minutes; p < 0.001). This finding aligns with prior reports attributing longer laparoscopic operative durations to factors such as increased setup time for laparoscopic equipment, the learning curve associated with advanced suturing techniques, and the technical intricacies of morcellation and specimen retrieval [12,17]. However, the slightly prolonged operative time in LM did not appear to negatively impact patient recovery.

Indeed, LM demonstrated superior postoperative outcomes compared to OM, including lower postoperative pain scores, reduced analgesic requirements, shorter hospital stays, and quicker returns to normal daily activities [18,19]. These advantages highlight the minimally invasive nature of LM and its growing role in gynecologic surgery. Moreover, recent advances, such as in-bag transvaginal extraction of fibroids and the introduction of radiofrequency ablation, have expanded the feasibility and safety of LM for larger fibroids, potentially narrowing the traditional size-based indications for OM [14,15].

An additional factor worth noting is the relationship between age and postoperative pain. A systematic review demonstrated a negative correlation between patient age and reported pain intensity, suggesting that younger women tend to experience higher pain scores and require more postoperative analgesia compared to older counterparts [20]. This finding may partially explain variations in postoperative analgesic use across age groups in our study and warrants further exploration in future research.

Limitations

First, the prospective but non-randomized design restricts the ability to establish causal relationships. Second, allocation was non-randomized, with patients having larger or multiple fibroids often directed toward OM based on clinical and surgical considerations; thus, selection bias and potential confounding could limit the internal validity of direct comparisons between groups. Third, the sample size, while adequate for primary outcomes, was insufficient to assess rare complications or long-term fertility outcomes. Fourth, pain assessment, despite standardized tools and blinded evaluators, remained inherently subjective. Fifth, the relatively short follow-up period prevented evaluation of fibroid recurrence, obstetric outcomes, and long-term patient satisfaction. Finally, the single-center nature of the study and possible unmeasured confounders, such as surgeon expertise or patient comorbidities, may limit the generalizability of the findings.

## Conclusions

This study demonstrates that LM offers significant advantages over OM, including reduced intraoperative blood loss, lower postoperative pain, shorter hospital stays, and quicker returns to normal activities, despite a longer operative time. The size of fibroids was identified as a key factor influencing the choice of surgical approach, with larger fibroids more likely to necessitate open surgery. These findings support the increased adoption of minimally invasive techniques for appropriately selected patients. Nonetheless, individualized surgical planning that takes into account fibroid characteristics, surgeon expertise, and patient preferences is crucial. Future research involving larger cohorts and long-term follow-up is needed to evaluate recurrence rates, fertility outcomes, and patient-reported quality of life associated with different surgical methods.
